# Quality assessment of evidence-based clinical practice guidelines for the management of pregnant women with sickle cell disease using the AGREE II instrument: a systematic review

**DOI:** 10.1186/s12884-020-03241-y

**Published:** 2020-10-07

**Authors:** Yasser S. Amer, Yasser Sabr, Ghada M. ElGohary, Amer M. Altaki, Osamah T. Khojah, Ahmed El-Malky, Musa F. Alzahrani

**Affiliations:** 1 Clinical Practice Guidelines Unit, Quality Management Department, King Saud University Medical City, King Saud University, Riyadh, Saudi Arabia; 2 Department of Pediatrics, King Khalid University Hospital, King Saud University Medical City, King Saud University, Riyadh, Saudi Arabia; 3 Research Chair for Evidence-Based Health Care and Knowledge Translation, Deanship of Scientific Research, King Saud University, Riyadh, Saudi Arabia; 4 Alexandria Center for Evidence-Based Clinical Practice Guidelines, Alexandria University, Alexandria, Egypt; 5 Department of Obstetrics and Gynecology, College of Medicine, King Saud University, Riyadh, Saudi Arabia; 6 Prince Sattam Bin Abdul Aziz Research Chair for Epidemiology and Public Health, College of Medicine, King Saud University, Riyadh, Saudi Arabia; 7 University Oncology Center, King Saud University Medical City, College of Medicine, King Saud University, Riyadh, Saudi Arabia; 8 Internal Medicine and Clinical Hematology, Faculty of Medicine, Ain Shams University, Cairo, Egypt; 9 Pathology Department, College of Medicine, King Saud University, Riyadh, Saudi Arabia; 10 Morbidity and Mortality Unit, King Saud University Medical City, King Saud University, Riyadh, Saudi Arabia; 11 Public Health and Community Medicine Department, Theodor Bilharz Research Institute (TBRI), Academy of Scientific Research, Cairo, Egypt; 12 Department of Medicine, College of Medicine, King Saud University, Riyadh, Saudi Arabia

**Keywords:** Sickle cell disease, pregnancy, practice guidelines, AGREE II instrument, quality assessment

## Abstract

**Background:**

The management of pregnant women with sickle cell disease (SCD) poses a major challenge for maternal healthcare services owing to the potential for complications associated with morbidity and mortality. Trustworthy evidence-based clinical practice guidelines (CPGs) have a major impact on the positive outcomes of appropriate healthcare. The objective of this study was to critically appraise the quality of recent CPGs for SCD in pregnant women.

**Methods:**

Clinical questions were identified and the relevant CPG and bibliographic databases were searched and screened for eligible CPGs. Each CPG was appraised by four independent appraisers using the AGREE II Instrument. Inter-rater analysis was conducted.

**Results:**

Four eligible CPGs were appraised: American College of Obstetricians and Gynecologists (ACOG), National Heart, Lung, and Blood Institute (NHLBI), National Institute of Health and Care Excellence (NICE), and Royal College of Obstetricians and Gynaecologists (RCOG). Among them, the overall assessments of three CPGs (NICE, RCOG, NHLBI) scored greater than 70%; these findings were consistent with the high scores in the six domains of AGREE II, including:[1] scope and purpose,[2] stakeholder involvement,[3] rigor of development,[4] clarity of presentation,[5] applicability, and [6] editorial independence domains. Domain [3] scored (90%, 73%, 71%), domain [5] (90%, 46%, 47%), and domain [6] (71%, 77%, 52%) for NICE, RCOG, and NHLBI, respectively. Overall, the clinical recommendations were not significantly different between the included CPGs.

**Conclusions:**

Three evidence-based CPGs presented superior methodological quality. NICE demonstrated the highest quality followed by RCOG and NHLBI and all three CPGs were recommended for use in practice.

## Background

Sickle cell disease (SCD) is a genetic disorder that causes a vaso-occlusive phenomenon and hemolysis along with a myriad of other major complications that could be life-threatening. It is one of the commonest inherited diseases, worldwide, and is inherited as an autosomal recessive disease due to the substitution of valine for glutamic acid at the sixth amino acid of the beta-globin chain [1]. This substitution leads to the production of a hemoglobin variant that is poorly soluble when deoxygenated. The clinical features, which include a vaso-occlusive crisis, are the result of the polymerization of the deoxygenated hemoglobin S. SCD is associated with significant maternal morbidity and mortality in pregnant women. The recognized complications include maternal mortality, preeclampsia, eclampsia, venous thromboembolism, cesarean delivery, intrauterine fetal death, and fetal growth restriction [2].

The prevalence of SCD varies among countries. For example, data from the United States showed that the overall prevalence is roughly about 4.83 per 10,000 deliveries; [3] 28.5% of women with SCD develop a crisis at the time of delivery. The maternal mortality rate was reported to be 1.6 per 1000 deliveries in women with SCD compared to 0.1 per 1000 deliveries in those without SCD [3].

Information about the prevalence of SCD in Saudi Arabia is limited and different among the various provinces, with the highest prevalence reported in the Eastern province followed by the Southwest province [4].

SCD in pregnancy tends to cause higher episodes of painful crises and a higher frequency of blood transfusion [2]. Although the complications of SCD are more commonly associated with the HbSS genotype, patients with other types of SCD such as sickle-beta thalassemia (HbSC genotype) and Hemoglobin SC disease (HbSC genotype) should receive the same level of care as those with HbSS. The development of a multidisciplinary care approach and comprehensive sickle cell centers seem to be associated with a decrease in the incidence of perinatal complications [5, 6].

Clinical Practice Guidelines (CPGs) were defined by the Health and Medicine Division of the American National Academies (formerly, the Institute of Medicine [IOM]), as “statements that include recommendations intended to optimize patient care and are informed by a systematic review of evidence and an assessment of the benefits and harms of alternative care options” [7]. To date, there are no national CPGs for the management of SCD in pregnant women in Saudi Arabia.

The second edition of the Appraisal of Guidelines for Research and Evaluation Instrument (AGREE II) is the gold standard for the quality assessment or critical appraisal of CPGs. It was first published in its original form in 2003 and lastly updated in 2017 by the AGREE enterprise. AGREE II is a validated quantitative tool that has been cited in more than 1013 articles and endorsed by several healthcare organizations [8, 9]. AGREE II identifies constituents that must be addressed by CPGs to improve their quality, and henceforth, ensure their expected trustworthiness, and positive impact on healthcare outcomes [9].

The objective of this study was to conduct a systematic review and critically appraise the quality of recently published CPGs for SCD in pregnancy using the AGREE II instrument as a part of the CPG adaptation program [10].

## Methods

The protocol for this study is published in the International Prospective Register of Systematic Reviews (PROSPERO; https://www.crd.york.ac.uk/PROSPERO/display_record.php?RecordID=145443) [11].

### Inclusion and exclusion criteria

Three reviewers independently reviewed the retrieved CPGs, based on the following inclusion criteria: evidence-based with clear and detailed documentation of the development methods; available in the English language; obtained from original sources (de novo developed); had a national or international scope; published, amended or updated between January 1, 2014, and December 31, 2018 (the search was repeated before the submission of the final manuscript to identify new relevant CPGs; those published over the last five years only (2014–2018) were included in this study (because the period between two updates was reported to range from two to five years, in several CPG handbooks). CPGs published by an organization or having group authorship in a CPG database or peer-reviewed journal were also included in this study. Only the most current version of each Source CPG was included [11, 12].

The exclusion criteria included CPGs that were published earlier than 2014, written in a non-English language, adapted from other Source CPG (s), presented as consensus or expert-based statements, and written by a single author [11].

### Search strategy and selection of SCD in pregnancy CPGs

Literature searches of bibliographic databases (Medline/PubMed and Google Scholar), EBSCO DynaMed Plus (USA) and relevant CPG databases, such as the ECRI Institute Guidelines Trust, National Institute of Health and Care Excellence (NICE; UK), Guidelines International Network (G-I-N) International guideline library, Scottish Intercollegiate Guidelines Network (SIGN; UK), and the Australian National Health and Medical Research Council (NHMRC; Australia) were performed. Moreover, we searched databases of national and international societies specializing in fields related to the topic of SCD in pregnancy, such as the American College of Obstetricians and Gynecologists (ACOG), Royal College of Obstetricians and Gynaecologists (RCOG), Royal Australian and New Zealand College of Obstetricians and Gynaecologists (RANZCOG), Society for Maternal Fetal Medicine (SMFM), Saudi Society for Obstetrics and Gynecology (SSOG), and Arab Association of Obstetrics and Gynaecology Societies (FAGOS). The keywords used were “sickle cell disease” AND “pregnancy” OR “pregnant women” AND “guideline,” “practice guideline,” “clinical practice guideline,” “practice parameter,” “guidance,” OR “recommendations” [11].

The PubMed electronic search strategy included the following: “anemia, sickle cell“[MeSH Terms] OR (“anemia“[Title] AND “sickle“[Title] AND “cell“[Title]) OR “sickle cell anemia“[Title] OR (“sickle“[Title] AND “cell“[Title] AND “disease“[Title]) OR “sickle cell disease“[Title]) AND “pregnan“[Title] AND (“pregnancy“[MeSH Terms] OR “pregnancy“[Title]) OR (“pregnant women“[MeSH Terms] OR (“pregnant“[Title] AND “women“[Title]) OR “pregnant women“[Title]) AND (“guideline“[Publication Type] OR “guidelines as topic“[MeSH Terms] OR “guidelines“[Title]) AND (Practice Guideline [ptyp] AND (“2014/01/01“[PDAT] : “2019/12/31“[PDAT]) AND “humans“[MeSH Terms]) AND (practiceguideline [Filter]). Furthermore, the PIPOH (Patient Population, Interventions, Professionals, Outcomes, and Healthcare Setting) model was used to support the CPG eligibility process [10]. Three reviewers (YA, MA, YS) independently screened titles and abstracts of the retrieved CPGs and articles that met the inclusion criteria. The screening was re-checked by three different reviewers (GE, AA, OK). Disagreements were resolved by focus group discussions after retrieval and review of the full-text articles or full CPG documents, including links to any accessible online supplementary documents or web resources. The search was repeated before the submission of the final manuscript to retrieve any new eligible CPG.

### Assessment of CPGs using the AGREE II instrument

The AGREE II instrument (www.agreetrust.org) consists of 23 items organized in 6 domains: scope and purpose, stakeholder involvement, rigor of development, clarity of presentation, applicability, and editorial independence [13]. Each item is scored on a Likert scale (1–7). The AGREE II evaluation was guided by utilizing its online version, “My AGREE PLUS,” which supports the presence of a CPG “appraisal group” for each CPG that compiles and calculates the ratings of the items into domain ratings, and comments [13]. The four AGREE II appraisers in the study had the relevant clinical expertise in obstetrics and gynecology (YS, AA), internal medicine (GE, MA), and hematology (GE, MA); furthermore, an expert CPG methodologist (YA) was included. At the outset, the CPG methodologist conducted capacity building sessions for the reviewers via hands-on sessions on the concepts, evidence-based CPG standards, and use of the AGREE II instrument. Each reviewer scored his/her assigned CPGs, and the included CPGs was critically appraised by all the five reviewers. The CPG documents, including any updates, plus any relevant supplementary information or links to online webpages related to the CPG methods or CPG implementation tools were reviewed in full by all the appraisers. For each item, the AGREE appraisers were asked to record the justifications for their scores in the “Comments” section. Wide discrepancies between the assessors’ scores for items or questions (a difference of more than 3 between the scores) were resolved by asking those who had provided the outlying scores to re-assess the questions after discussions with the entire group. The standardized AGREE domain scores or ratings (%) were automatically calculated using My AGREE PLUS. A cut-off point of 70% was set for each AGREE standardized domain score or rating. After the appraisal, we focused on the scores of domains 3 and 5 to facilitate the filtration and final evaluation of the reporting quality of the included CPGs. Similar cut-off values have been reported in previous studies [14, 15]. In addition to the classification of the six AGREE II domains, the evidence-bases of the included CPGs and their references sections were screened for systematic reviews or meta-analyses, specifically for Cochrane reviews. We utilized the PRISMA statement flow diagram and checklist for reporting our review [16–18]. There was no patient nor public involvement in this study.

### Inter-rater analysis

Inter-rater reliability assessment tests (IRR) were conducted to determine the agreement levels between the raters. Percent agreement IRR was used for every question (or item) in each standardized domain in the four CPGs included in this study to assess the level of agreement among the four raters and the percent agreement of the first overall assessment (OA1) of the AGREE II instrument. Furthermore, the Intra-class correlation coefficient (ICC) was used to measure the consistency in the ratings or capacities of the datasets that were gathered as clusters or arranged into clusters, including the second overall assessment (OA2 or “recommend this CPG for use”). ICC is one of the most prevalent IRR approaches used when the number of raters is more than two. A high ICC (or Kappa; K) value (near 1) indicated a high resemblance between standards from the same set. A low Kappa value (near zero) indicated that the standards from the same set are not alike. One-Way Random analysis of variance was used SPSS Statistics, version 21 was used to analyze the data in this study. The ICC was used due to the diversity in the numerical data obtained from the groups or clusters. It helped us in detecting the reproducibility of the results and in determining how closely the peers resembled each other with regard to certain traits or characteristics. The agreement between two ordinal scale classifications was evaluated, and weighted Kappa (Quadratic Weights) was used because the data was obtained from an ordered scale.

Linear weights were used because the difference between the first and second categories had the same importance as that between the second and third categories, and so on. The agreement was quantified using the K statistic, [19, 20] where K = 1 when there is perfect agreement between the classification systems, K = 0 when there is no agreement better than chance, and K is negative when the agreement is worse than chance. The strength of the agreement based on the K value was classified as follows: < 0.20 (poor), 0.21–0.30 (fair), 0.31–0.40 (moderate), 0.41–0.60 (good), 0.61–0.80 (very good), and 0.81–1.00 (excellent) [21].

## Results

### Identification of SCD in pregnancy CPGs

**Fig. 1:**
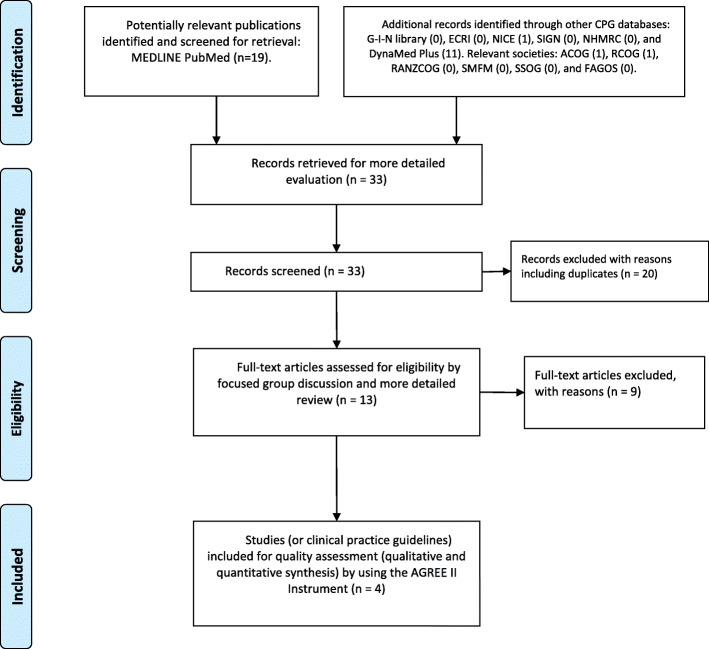
PRISMA flow diagram. Systematically searching and selecting the clinical practice guidelines for the management of pregnant women with sickle cell disease. From: Moher D, Liberati A, Tetzlaff J, Altman DG, The PRISMA Group (2009). Preferred Reporting Items for Systematic Reviews and Meta-Analyses: The PRISMA Statement. PLoS Med 6(7): e1000097. doi:10.1371/journal.pmed1000097. For more information, visit www.prisma-statement.org

The results of the search in the PRISMA statement flow diagram are shown in Fig. 1 [17, 18]. The initial list of 33 records was reviewed and filtered by the assessors. Among them, 29 were excluded because they did not meet the inclusion criteria. The four recent SCDs in pregnancy CPGs that complied with our PIPOH and inclusion criteria were shown in Fig. 1. The CPGs were developed by ACOG in January 2007 (reaffirmed in 2018) [22], NICE in June 2012 (with a minor update in August 2016) [23], RCOG in August 2011 (updated in May 2018) [24], and the US Department of Health and Human Services, Public Health Service, National Institutes of Health, National Heart, Lung, and Blood Institute (NHLBI) in 2014 [25]

.

### Key characteristics of SCD in pregnancy CPGs

Table 1 highlights the characteristics of all the eligible CPGs; two CPGs were developed by US-based (*n* = 2, 50%), and two CPGs (*n* = 2, 50%) by UK-based organizations. The four CPGs were developed by two reference specialized professional organizations (ACOG, RCOG), and two national evidence-based healthcare improvement organizations (NICE, NHLBI). All the organizations are from high-income countries [22–25].

**Table 1:** Characteristics of the included CPGs

Title	Year of publication	Country	Level of development	Organization (short name)	Total number of references
**ACOG Practice Bulletin Clinical Management Guidelines for Obstetrician-Gynecologists**, **Number 78, January 2007 Hemoglobinopathies in Pregnancy** [22]	2007 (Reaffirmed 2018)	United States	National	American College of Obstetricians and Gynecologists (ACOG)	26 (one NCSR)
**Sickle cell disease: managing acute painful episodes in the hospital. NICE Clinical guideline 143** [23]	2012 (minor update in 2016)	United Kingdom	National	National Institute of Health and Care Excellence (NICE)	97 (one NCSR) (reviewed and excluded NCSR and CSRs were not counted)
**Management of Sickle Cell Disease in Pregnancy (Green-top Guideline No. 61). Royal College of Obstetricians and Gynaecologists** [24]	2011 (updated 2018)	United Kingdom	National	Royal College of Obstetricians and Gynaecologists (RCOG)	80 (one NCSR, 5 CSR)
**Evidence-Based Management of Sickle Cell Disease: Expert Panel Report (EPR), 2014** [25]	2014 (update)	United States	National	US Department of Health and Human Services, National Institutes of Health, National Heart, Lung, and Blood Institute (NHLBI)	428 (5 NCSR, one CSR)

**Abbreviations**: *CPG* clinical practice guideline; *CSR* Cochrane systematic review; *NCSR* Non-Cochrane systematic review

### Reporting the quality of SCD in pregnancy CPGs

The standardized domain ratings of AGREE II are summarized in Table 2, and the appraisers’ comments are presented in Table 3.

**Table 2:** AGREE II standardized domain scores for the included CPGs

CPGs/ AGREE II Domains-standardized scores (%)	ACOG 2018[22]	NICE 2016[23]	RCOG 2018[24]	NHLBI 2014[25]
**Domain 1. Scope and purpose** **Items 1–3**: Objectives; Health question(s); Population (patients, public, etc.).	76%	93%	89%	88%
**Domain 2. Stakeholder Involvement** **Items 4–6**: Group Membership; Target population preferences and views; Target users	33%	85%	76%	64%
**Domain 3. Rigor of development** **Items 7–14**: Search methods; Evidence selection criteria; Strengths and limitations of the evidence; Formulation of recommendations; Consideration of benefits and harms; Link between recommendations and evidence; External review; Updating procedure	**41%**	**90%**	**73%**	**71%**
**Domain 4. Clarity and presentation** **Items 15–17**: Specific and unambiguous recommendations; Management options; Identifiable key recommendations	63%	89%	83%	83%
**Domain 5. Applicability** **Items 18–21**: Facilitators and barriers to application; Implementation advice/ tools; Resource implications; Monitoring/ auditing criteria	**24%**	**90%**	**46%**	**47%**
**Domain 6. Editorial independence** **Items 22, 23**: Funding body; Competing interests	19%	71%	77%	52%
**Overall Assessment 1** (Overall quality)	46%	83%	79%	83%
**Overall Assessment 2** (Recommend the CPG for use by the four appraisers)	Yes (n = 1); Yes with modifications (n = 2); No (n = 1).	Yes (n = 1); Yes with modifications (n = 3); No (n = 0).	Yes (n = 2); Yes with modifications (n = 2); No (n = 0).	Yes (n = 3); Yes with modifications (n = 1); No (n = 0).

**Abbreviations**: *ACOG* American College of Obstetricians and Gynecologists; *AGREE II* Appraisal of Guidelines for Research and Evaluation II; *CPG* clinical practice guideline or guidance; *NICE* National Institute of Health and Care Excellence; *NHLBI* National Institutes of Health, National Heart, Lung, and Blood Institute; and *RCOG* Royal College of Obstetricians and Gynaecologists

**Table 3:** Reviewers’ comments on the four CPGs organized according to the standardized domains in AGREE II 22–25*

AGREE II Domain	Strengths	Limitations
**Domain 1. Scope and purpose**	• Objectives, purpose, health intent, clinical questions, and patient population were clearly mentioned in the CPG full document or the website using the PICO model (NICE, NHLBI, RCOG).	• Target users were general rather than specific (ACOG)
**Domain 2. Stakeholder Involvement**	• GDG members’ names, specialties, institutions, and geographical locations were clearly mentioned and easy to find. GDG included methodologist(s) (NICE, RCOG). • GDG included members from relevant professional groups including patient representatives (NICE).	• GDG disciplines and roles were not clearly mentioned (ACOG). • GDG was missing some key disciplines (e.g. pharmacists and nurses) (RCOG). • Lack of adequate and clear descriptions of patient participation or preferences and target users (ACOG, NHLBI).
**Domain 3. Rigor of development**	• Detailed evidence search keywords were mentioned (NICE, RCOG). • The GRADE (Grading of Recommendations Assessment, Development and Evaluation) approach to assess the quality of evidence was utilized (NICE, NHLBI). • Recommendations include health benefits, harms, and side effects of recommendations with or without a discussion of their trade-offs (NICE, NHLBI). • All recommendations were linked to their relevant primary source of evidence (NICE, NHLBI, RCOG). • Lists and processes of external review were clearly reported and easy to find (NICE, NHLBI, RCOG). • Updating was clearly mentioned (NICE, RCOG).	• Lack of detailed search strategy (ACOG). • Strengths and limitations of the body of evidence (evidence tables) were not clearly reported (ACOG). • Lack of detailed process for formulation of the recommendations, and discussion of a trade-off between harms and benefits (ACOG, RCOG). • Details and methods of the external review process and outcomes were not clearly reported (ACOG). • Review and update process was not reported (ACOG, NHLBI).
**Domain 4. Clarity and presentation**	• This domain was well-addressed in most included CPGs, where key recommendations were specific, unambiguous, and easily identifiable in all CPGs (NICE, NHLBI, RCOG).	• Management of SCD Crisis in different pregnancy trimesters and abnormal fetal surveillance management were not highlighted (ACOG).
**Domain 5. Applicability**	• Some facilitators and barriers to implementations and clinical governance issues were discussed (NHLBI, NICE, RCOG). • A package of CPG Implementation tools was provided like educational tools (NICE), protocols (NHLBI), summary document (NHLBI, NICE, RCOG), patient information (NHLBI, NICE), clinical algorithm or pathway (NHLBI, NICE), baseline assessment sheet (NICE), Mobile App (RCOG). • Quality standards, measures, indicators, and/ or clinical audit criteria were provided (NICE, RCOG). • A formal economic analysis was conducted (NICE).	• Facilitators and barriers to implementations were not explicitly mentioned (ACOG). • Implementation tools were not provided (ACOG). • Quality measures or key performance indicators were not provided (ACOG, NHLBI). • No formal economic analysis was conducted (ACOG, NHLBI, and RCOG).
**Domain 6. Editorial independence**	• Funding with or without an influence statement was mentioned (NICE, NHLBI, RCOG). • DCOI statements were clearly provided (NICE, NHLBI, RCOG).	• Funding and influence statements were not clearly reported (ACOG, NHLBI). • No DCOI statements were provided (ACOG).

**Abbreviations**: *ACOG* American College of Obstetricians and Gynecologists; *AGREE II* Appraisal of Guidelines for Research and Evaluation II; *CPG* clinical practice guideline or guidance; *DCOI* declaration of conflict of interests; *NICE* National Institute of Health and Care Excellence; *NHLBI* National Institutes of Health, National Heart, Lung, and Blood Institute; *PICO* patient population -intervention(s)-comparison(s)-outcome(s); and *RCOG* Royal College of Obstetricians and Gynaecologists. *Comments specific to certain CPG(s) were indicated by parentheses

#### Domain 1: Scope and purpose

The AGREE II standardized score for domain 1 ranged from 76–93%. The scores of three CPGs were greater than 70% in domain 1 (NICE-2012, 93%; RCOG-2018, 89%; and NHLBI-2017, 88%).

#### Domain 2: Stakeholder involvement

The standardized scores for domain 2 ranged from 33–85%. The scores of two CPGs were greater than 70% (NICE-2016, 85% and RCOG-2018, 76%).

#### Domain 3: Rigor of development

The standardized scores for domain 3 ranged from 41–90%; the scores of three CPGs were greater than 70% (NICE-2016, 90%; RCOG-2018, 73%; and NHLBI-2017, 71%).

#### Domain 4: Clarity of presentation

The standardized scores for domain 4 ranged from 63% to 89%, and the cores of three CPGs were greater than 70% (NICE-2016, 89%; RCOG-2018, 83%; and NHLBI-2017, 83%).

#### Domain 5: Applicability

The standardized scores for domain 5 ranged from 24–90%. Only one CPG scored greater than 70% in this domain (NICE-2016 = 90%).

#### Domain 6: Editorial independence

The standardized scores for domain 6 ranged from 19–77%. The scores of two CPGs were greater than 70% (RCOG-2018, 76% and NICE-2016, 85%).

#### Overall assessment

**Fig. 2:**
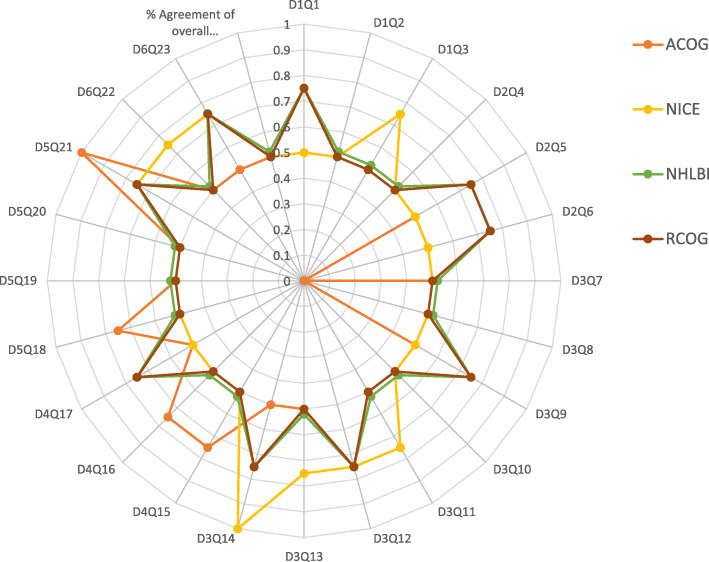
Using a Radar chart to map the AGREE II 23-questions, 6-domains, and the first overall assessment for eligible appraised clinical guidelines. Abbreviations: ACOG: American College of Obstetricians and Gynecologists, AGREE: Appraisal of Guidelines for Research and Evaluation, CPG: clinical practice guideline or guidance, D: AGREE II Domain, NICE: National Institute of Health and Care Excellence, NHLBI: National Institutes of Health, National Heart, Lung, and Blood Institute, Q: AGREE II Question (or Item), RCOG: Royal College of Obstetricians and Gynaecologists, and SCD: Sickle cell disease.

**Fig. 3:**
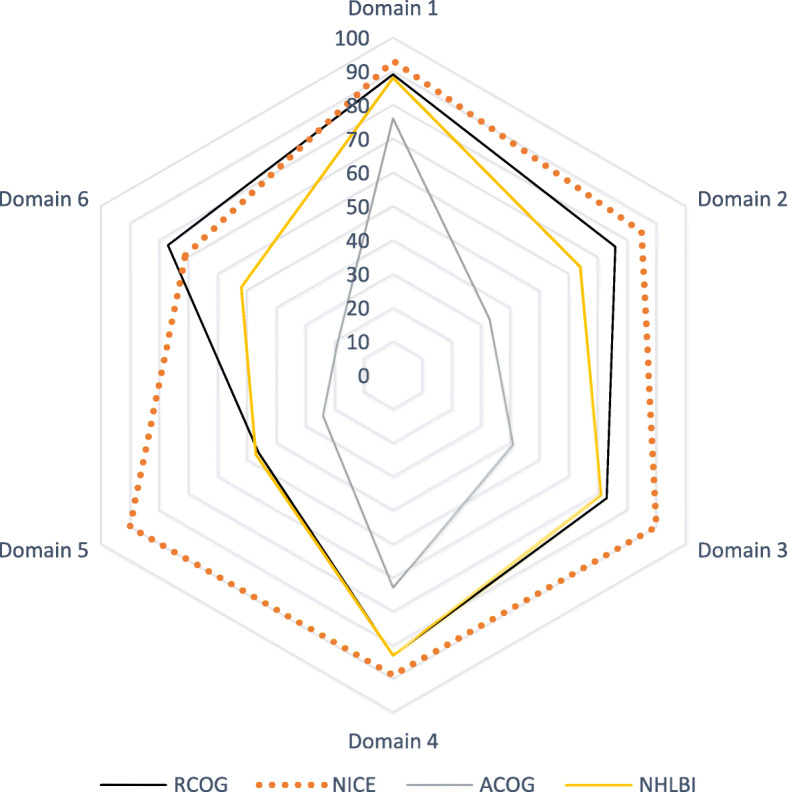
Radar map of the AGREE II final standardized domain scores for eligible appraised clinical guidelines. Abbreviations: ACOG: American College of Obstetricians and Gynecologists, AGREE: Appraisal of Guidelines for Research and Evaluation, CPG: clinical practice guideline or guidance, NICE: National Institute of Health and Care Excellence, NHLBI: National Institutes of Health, National Heart, Lung, and Blood Institute, OA1: AGREE II overall assessment 1, RCOG: Royal College of Obstetricians and Gynaecologists, and SCD: Sickle cell disease.

The AGREE II standardized domain scores for the first overall assessment ranged from 46–83%. Three CPGs scored greater than 70% (NHLBI, NICE, RCOG), consistent with their high scores in the six domains. The calculated AGREE II domain scores are shown in Figs. 2 and 3. The radar maps illustrate the final scores, expressed as percentages, for every included CPG in each of the six domains in Fig. 2 and each of the 23 questions in Fig. 3. The higher standardized domain scores are mapped toward the periphery (closer to 100%) and lower domain scores are plotted toward the center. The graphs illustrate a visual display of the relative strengths or weaknesses of each CPG by domain, question, and OA1 when compared to the other plotted CPGs.

### Recommending the CPGs for SCD in pregnancy for use in practice

The second (overall) assessment with regard to the recommendation for the use of the CPG in practice revealed a consensus between the reviewers. Two of the appraised CPGs were recommended for use without modification (RCOG and NHLBI), while the other two were recommended for use with modifications (NICE and ACOG).

The strengths and limitations of the included CPGs are summarized in Table 3, based on consensus and the comments of the CPG appraisers for each item in AGREE II. All four CPGs cited systematic reviews in their references list. The largest number of systematic reviews were cited in the RCOG CPG (n = 6), and five (83%) of them were Cochrane reviews. Overall, the different options of care in the management of SCD in pregnant women were similar in these CPGs (Table 4).

**Table 4:** Summary of key recommendations in the four CPGs from ACOG, NHLBI, NICE, and RCOG*

CPGs/ Recommendations	ACOG [22]	NHLBI [25]	NICE [23]	RCOG [24]
**Preconception care**				
**Genetic screening**	Individuals of African, Southeast Asian, and Mediterranean descent are at increased risk for being carriers of hemoglobinopathies and should be offered carrier screening and, if both parents are determined to be carriers, genetic counseling.	• If the partner of a man or woman with SCD has unknown SCD or thalassemia status, refer the partner for hemoglobinopathy screening. • After testing, refer couples who are at risk for having a potentially affected fetus and neonate for genetic counseling.	Not Mentioned	Women and men with SCD should be encouraged to have the hemoglobinopathy status of their partner determined before they embark on pregnancy. If identified as an “at risk couple,” as per National Screening Committee guidance, they should receive counseling and advice about reproductive options.
**Penicillin prophylaxis**	Not mentioned	Mentioned for the pediatric but not for the pregnant women population.	Not mentioned	Penicillin prophylaxis or the equivalent should be prescribed
**Vaccination status updated pre-pregnancy**	Not Mentioned	Mentioned in general for the adult population but not specifically for pregnant women	Not Mentioned	Women should be given *H. influenza* type b and the conjugated meningococcal C vaccine as a single dose, if they have not received it as part of primary vaccination. The pneumococcal vaccine should be given every 5 years. Hepatitis B vaccination is recommended and the woman’s immune status should be determined pre-conceptually. Women with SCD should be advised to receive influenza and “swine flu” vaccines, annually.
**Vitamin supplementation**	Pregnant patients with SCD need increased prenatal folic acid supplementation. The standard 1 mg of folate in prenatal vitamins is not adequate for patients with hemoglobinopathies; 4 mg per day of folic acid should be prescribed because of the continual turnover of red blood cells.	Folic acid supplementation should be used whenever considering or at risk of pregnancy to prevent neural tube defects.	Not Mentioned	Folic acid (5 mg) should be given once daily both pre-conceptually and throughout pregnancy.
**Medication review**	Hydroxyurea has been shown to reduce the frequency of painful crises in non-pregnant patients with severe SCD. However, the use of hydroxyurea is not recommended during pregnancy because it is teratogenic.	In females who are pregnant or breastfeeding, discontinue hydroxyurea therapy.	Not Mentioned	• Hydroxycarbamide (hydroxyurea) should be stopped at least 3 months before conception. • Angiotensin-converting enzyme inhibitors and angiotensin receptor blockers should be stopped before conception.
**Antenatal care**				
**Antenatal hemoglobinopathy screening**	Not mentioned	Not mentioned	Not Mentioned. But a link to the “NHS Sickle Cell and Thalassaemia Screening Program” was provided.	If the woman has not been seen pre-conceptually, she should be offered partner testing. If the partner is a carrier, appropriate counseling should be offered as early as possible in pregnancy – ideally by 10 weeks of gestation – to allow the option of first-trimester diagnosis and termination, based on the woman’s choice.
**Medications during pregnancy**	Not mentioned	Not clearly mentioned for women during pregnancy.	Mentioned under research recommendations	• If women have not undergone a pre-conceptual review, they should be advised to take folic acid (daily) and prophylactic antibiotics (if not contraindicated). Drugs that are unsafe in pregnancy should be stopped immediately. • Iron supplementation should be given only if there is laboratory evidence of iron deficiency. • Women with SCD should be considered for low-dose aspirin 75 mg once daily from 12 weeks of gestation to reduce the risk of developing pre-eclampsia. • Women with SCD should be advised to receive prophylactic low-molecular-weight heparin during antenatal hospital admissions.
**Blood transfusion or prophylactic exchange transfusion for pregnancies**	Recommended just to keep of Hb S to approximately 40% While simultaneously raising the total hemoglobin concentration to about 10 g/dL.	Not Mentioned	Not Mentioned	• Routine prophylactic transfusion is not recommended during pregnancy for women with SCD. • If acute exchange transfusion is required for the treatment of a sickle complication, it may be appropriate to continue the transfusion regimen for the remainder of the pregnancy. • Blood should be matched for an extended phenotype including full rhesus typing (C, D, and E) as well as Kell typing. • Blood used for transfusion in pregnancy should be cytomegalovirus negative.
**Ultrasound Scanning and fetal surveillance during pregnancy**	Pregnancies in women with sickle cell disease are at increased risk for spontaneous abortion, preterm labor, IUGR, and stillbirth. For this reason, a plan for serial ultrasound examinations and antepartum fetal testing is reasonable.	Fetal surveillance, which includes growth ultrasounds and antepartum testing (non-stress tests, biophysical profiles, and contraction stress tests), may lead to planned early delivery and can reduce but not eliminate risks (not mentioned as a recommendation).	Not mentioned	• Women should be offered a viability scan at 7–9 weeks of gestation. Women should be offered the routine first-trimester scan (11–14 weeks of gestation) and a detailed anomaly scan at 20 weeks of gestation. In addition, women should be offered serial fetal biometry scans (growth scans) every 4 weeks from 24 weeks of gestation.
**Acute painful crisis**	Major complications (e.g., worsening anemia; intrapartum complications such as hemorrhage, septicemia, and cesarean delivery; painful crisis; and chest syndrome) may require intervention with an exchange transfusion (not mentioned as a recommendation). Painful crises in pregnancy as well as in the non-pregnant patients are managed by rapid assessment of the level of pain and prompt administration of analgesia.	Not clearly mentioned for women during pregnancy.	• For pregnant women with an acute painful sickle cell episode, seek advice from the obstetrics team and refer when indicated. • Offer all patients regular paracetamol and NSAIDs (non-steroidal anti-inflammatory drugs) by a suitable administration route, in addition to an opioid, unless contraindicated (Not clearly mentioned for women during pregnancy). • The use of NSAIDs should be avoided during pregnancy unless the potential benefits outweigh the risks. NSAIDs should be avoided for treating an acute painful sickle cell episode in women in the third trimester. See the “British National Formulary” for details of contraindications.	• Women with SCD who become unwell should have sickle cell crisis excluded as a matter of urgency. • Pregnant women presenting with acute painful crisis should be rapidly assessed by the multidisciplinary team and appropriate analgesia should be administered. Pethidine should not be used because of the associated risk of seizures. • Women admitted with sickle cell crisis should be looked after by the multidisciplinary team, involving obstetricians, midwives, hematologists, and anesthetists. • The requirement for fluids and oxygen should be assessed, and fluids and oxygen administered if required. • Thromboprophylaxis should be given to women admitted to the hospital with an acute painful crisis.
**Intrapartum care**				
**Timing and mode of delivery**	Not mentioned	Not mentioned	Not mentioned	• Pregnant women with SCD who have a normally growing fetus should be offered elective birth through induction of labor, or by elective cesarean section if indicated, after 38 + 0 weeks of gestation. • SCD should not in itself be considered a contraindication to attempting vaginal delivery or vaginal birth after cesarean section. • Blood should be cross-matched for delivery if there are atypical antibodies present (since this may delay the availability of blood), otherwise a “group and save” will suffice. • In women who have hip replacements (because of avascular necrosis) it is important to discuss suitable positions for delivery.
**Optimal mode of analgesia and anesthesia**	Not mentioned (analgesia mentioned with painful crisis).	Not clearly mentioned for women during pregnancy.	Not mentioned	• Women with SCD should be offered an anesthetic assessment in the third trimester of pregnancy. • Avoid the use of pethidine, but other opiates can be used. • Regional analgesia is recommended for cesarean section.
**Postpartum care**				
**Neonatal screening**	Not mentioned	Not mentioned	Not mentioned	In pregnant women where the baby is at high risk of SCD (i.e. the partner is a carrier or affected), early testing for SCD should be offered. Capillary samples should be sent to laboratories experienced in the routine analysis of SCD in newborn samples. This will usually be at a regional center.
**VTE prophylaxis**	Not Mentioned.	Not clearly mentioned for women during pregnancy.	Not mentioned	Low-molecular-weight heparin should be administered while in hospital and 7 days post-discharge following vaginal delivery or for a period of 6 weeks following cesarean section.
**Contraception**	Not Mentioned	• Progestin-only contraceptives (pills, injections, and implants), levonorgestrel IUDs, and barrier methods have no restrictions or concerns for use in women with SCD. • If the benefits are considered to outweigh the risks, combined hormonal contraceptives (pills, patches, and rings) may be used in women with SCD.	Not Mentioned	• Progestogen-containing contraceptives such as the progesterone-only pill, injectable contraceptives, and the levonorgestrel intrauterine system are safe and effective in SCD. • Estrogen-containing contraceptives should be used as second-line agents. • Barrier methods are as safe and effective in women with SCD as in the general population.

**Abbreviations**: *ACOG* American College of Obstetricians and Gynecologists; *CPG* clinical practice guideline or guidance; *DCOI* declaration of conflict of interests; *NICE* National Institute of Health and Care Excellence; *NHLBI* National Institutes of Health, National Heart, Lung, and Blood Institute; *PGD* Preimplantation genetic diagnosis; and *RCOG* Royal College of Obstetricians and Gynaecologists

### Inter-rater analysis

The results of the IRR tests showed a high strength of agreement for every question in every domain in the four CPGs among the four raters. As well as the percent agreement of the first overall assessment (OA1) in Fig. 2. Most of the K values were between 0.50 and 1.00, denoting good to excellent agreement. Only two evaluations (Fig. 3) pertaining to question 6 in domain 2 (D2Q6) and question 8 in domain 3 (D3Q8) revealed poor strengths of agreement (K = 0.0) in the ACOG CPG. As seen in Table 5, out of the 24 questions in the ACOG CPG, one demonstrated excellent agreement (K = 1), 16 showed good agreement (K = 0.5), 5 presented with very good agreement (K = 0.6–0.8), and two with poor agreement (K = 0.00); the results of the overall assessment (1) demonstrated good agreement (K = 0.5). In the RCOG 2011 evaluation, none of the 24 questions demonstrated excellent agreement, 15 presented with good agreement (K = 0.5), and 9 with very good agreement (K = 0.6–0.8); the overall assessment (1) showed good agreement (K = 0.5). In the NICE 2012 evaluation, one out of 24 questions demonstrated excellent agreement (K = 1), 16 presented with good agreement (K = 0.5), and seven with very good agreement (K = 0.6–0.8); the overall assessment (1) showed good agreement (K = 0.5). In the NHLBI evaluation, none of the 24 questions demonstrated excellent agreement, 15 presented with good agreement (K = 0.5), and 9 with very good agreement (K = 0.6–0.8); the overall assessment (1) showed good agreement (K = 0.5; Table 5). The results of the K value among the raters for the four guidelines with regard to the second overall assessment (OA2) revealed the following: number of observed agreements, 6 (37.50% of the observations); number of agreements expected by chance, 4.0 (25.00% of the observations); K = 0.167; standard error of K = 0.138 (95% confidence interval, − 0.103–0.437); and weighted K = 0.077.

**Table 5:** Classification of the strength of agreement among the four raters for the four clinical practice guidelines

	Poor	Fair	Good	Very good	Excellent	Overall assessment 1
**RCOG**[24]	0	0	15	9	0	Good
**NICE**[23]	0	0	16	7	1	Good
**ACOG**[22]	2	0	16	5	1	Good
**NHLBI**[25]	0	0	15	9	0	Good

**Abbreviations**: *ACOG* American College of Obstetricians and Gynecologists; *NICE* National Institute of Health and Care Excellence; *NHLBI* National Institutes of Health, National Heart, Lung, and Blood Institute; and *RCOG* Royal College of Obstetricians and Gynaecologists

## Discussion

To the best of our knowledge, this is the first study to systematically evaluate the quality of recently published CPGs for SCD in pregnancy using the AGREE II instrument.

Four CPGs addressing the management of pregnant women with SCD were assessed using the AGREE II instrument. Several areas of improvement in the methodological rigor of the included CPGs were highlighted. One CPG (ACOG) had significant gaps in its rigor of development (domain 3), which is the largest and core domain, and three CPGs demonstrated areas for improvement in their applicability (Domain 5). The weights of these two domains have been emphasized in this study. The NICE CPG received the highest reviewer agreement ratings [23]. All four CPGs included in this review had commonalities and differences in their clinical recommendations (Table 4). The common factors included genetic screening (ACOG, RCOG), genetic diagnosis (ACOG, NHLBI, RCOG), counseling during pregnancy (all four CPGs), transfusion or prophylactic exchange transfusion (ACOG, NICE, RCOG), fetal surveillance (ACOG, NHLBI, RCOG), and contraception (NHLBI, RCOG).

One discrepancy was observed in the form of a lack of clearly articulated recommendations for the vaccination status before pregnancy in three CPGs (ACOG, NHLBI, NICE). Two CPGs (ACOG and RCOG) were more specific about pregnancy when compared with the two other CPGs that contained general recommendations for SCD and smaller sections that focused on pregnancy. Out of the two specific CPGs, RCOG consistently presented with higher scores in all the domains. This systematic and objective assessment of the available CPGs is beneficial in supporting the decision to adopt or adapt the CPGs in clinical practice. After reviewing the four CPGs and given the appropriate rigor, consistently high scores, and clinical relevance of RCOG, we decided to adopt all the recommendations of this CPG in our clinical practice.

The findings of the current study revealed that the CPG assessment was accurate. There was excellent/ very good inter-rater agreement between the four assessors who evaluated the four CPGs using AGREE II. The proposed approach can be considered as a model for similar systematic reviews and quality assessments of CPGs.

Furthermore, the statistical analysis in this study illustrated the practicability of the AGREE II instrument as a valuable tool for the critical appraisal of CPGs, without compromising on the quality. Conceivably, inexperienced staff or non-professional reviewers would not have reached similar agreements with regard to the clinical features or characteristics in the CPGs, which could impact the judgment related to the provision of care to pregnant women with SCD.

In the first half of 2019, more than eight systematic critical appraisals of CPGs in obstetrics and gynecology were published using the AGREE II instrument. These included high priority health topics such as induction of labor[26], planned cesarean Sect. [27], recurrent pregnancy loss[28], packed red cells versus whole blood transfusion for severe pregnancy-related anemia and obstetric bleeding[29], gestational diabetes mellitus[30–32], and bladder pain syndrome/interstitial cystitis [33]. These studies identified several gaps, including differences, discrepancies, lack of evidence-base, and inconsistencies in some clinical recommendations among the CPGs; in addition, a few commonalities and similarities in some recommendations with advice to improve these variabilities were observed in CPGs [26–33].

### Strengths and limitations

One of the strengths of the current study is that the appraisal conducted in this review was performed by a specialized clinical team of obstetricians and gynecologists, internists, and hematologists guided by an expert CPG methodologist, which adds a layer of strength to the AGREE II assessment. The other advantages of this study include the following: (i) the use of an international, rigorously structured, and validated CPG appraisal tool: the AGREE II instrument; (ii) the appraisal of each CPG by four raters including three clinical topic experts and a CPG methodologist; (iii) a comprehensive search within several databases; and (iv) statistical assessment of the inter-rater differences.

Care providers for pregnant women with SCD must be encouraged to adopt and merge the principles of “evidence-based” and “eminence-based” healthcare in their daily practice through continuous training and education about the standards of high-quality CPGs and their appraisal tools [34–38]. The results of this review can be used as a basis for CPG development or adaptation for pregnant women with SCD. Furthermore, they highlight the importance of the inclusion of the AGREE II criteria during capacity building, which will aid the clinicians in identifying and adopting the CPGs for use in daily practice.

Our study also has several limitations. First, the AGREE II instrument has several updates and different versions. Some of the disadvantages of AGREE II have been addressed in the recently developed “AGREE-REX” (Recommendation EXcellence) tool that reports the clinical credibility of the CPG recommendations. AGREE-REX has been validated and shared publicly on the website [39]. The selection of 70% as a cut-off point for standard domain ratings is another potential limitation because the original AGREE II does not mandate such a cut- off; however, some studies have used this cut-off value [40]. Other limitations include the following: (i) the inclusion of English language CPGs only may have resulted in the exclusion of relevant CPGs intended for use in non-English speaking healthcare settings; (ii) this review mainly focused on CPGs for the management of pregnant women with SCD, due to its known burden and priority for maternity health, and did not evaluate other subcategories because it was out of the scope of this study; and (iii) the included CPGs belonged to two different healthcare systems (i.e. US-based and UK-based).

### Implications for practice: guidance for guideline uptake

The adaptation of CPGs has been identified as a valid and feasible alternative to de novo development, which is a resource-extensive process [41]. Evidence-based practice initiatives in some countries, especially those with low- and middle-income economies, have opted to utilize CPG adaptation rather than development [10]. Several formal adaptation methodologies are now available and could be further customized to local contexts [42]. Similar reviews like ours could inform relevant CPG adaptation or development projects for the SCD in pregnant women especially for groups with little experience, in using the AGREE II instrument.

The current critical appraisal highlights the importance of performing quality assessments of CPGs by clinicians to ensure the transparency and strength of the CPG development process according to international CPG standards and to support the provision of the best practice for pregnant women with SCD. We recommend incorporating the AGREE II appraisal of CPGs in the capacity building plans for obstetricians, gynecologists, and hematologists.

## Conclusions

The methodological qualities of three evidence-based CPGs were superior to that of the expert consensus. NICE followed by the RCOG and NHLBI SCD CPGs presented with the highest qualities and were recommended for use in practice. We recommend using the AGREE II criteria and the methodologies utilized in the RCOG and NICE CPGs as models.

## Data Availability

All data analyzed during this study are included in this published article.

## Abbreviations

ACOG: American College of Obstetricians and Gynecologists
AGREE: Appraisal of Guidelines for Research and Evaluation
CPG: clinical practice guideline or guidance
NICE: National Institute of Health and Care Excellence
NHLBI: National Institutes of Health, National Heart, Lung, and Blood Institute
RCOG: Royal College of Obstetricians and Gynaecologists
SCD: Sickle cell disease
